# The Association Between Maladaptive Metacognitive Beliefs and Emotional Distress in People Living With Amyotrophic Lateral Sclerosis

**DOI:** 10.3389/fpsyg.2021.609068

**Published:** 2021-02-26

**Authors:** Rachel Dodd, Peter L. Fisher, Selina Makin, Perry Moore, Mary Gemma Cherry

**Affiliations:** ^1^Institute of Population Health, University of Liverpool, Liverpool, United Kingdom; ^2^Liverpool University Hospitals NHS Foundation Trust, Liverpool, United Kingdom; ^3^Salford Royal NHS Foundation Trust, Salford, United Kingdom; ^4^Primary Care and Mental Health, University of Liverpool, Liverpool, United Kingdom

**Keywords:** amyotrophic lateral sclerosis, distress, metacognition, repetitive negative thinking, adults

## Abstract

**Objective:**

Approximately half of all people living with amyotrophic lateral sclerosis (ALS) experience persistent or recurrent emotional distress, yet little is known about the psychological processes that maintain emotional distress in this population. The self-regulatory executive functioning (S-REF) model specifies that maladaptive metacognitive beliefs and processes are central to the development and maintenance of emotional distress. This study explored whether maladaptive metacognitive beliefs are associated with emotional distress after controlling for demographic factors, time since diagnosis, and current level of physical functioning.

**Design:**

In a cross-sectional design, 75 adults with a diagnosis of ALS completed self-report questionnaires. Participants had a mean age of 60.40 years, mean duration of symptoms 63.92 months, and male:female gender ratio of 14:11.

**Main Outcome Measures:**

Questionnaires assessed emotional distress (HADS, adapted for ALS), physical functioning (ALSFRS-R), repetitive negative thinking (RTQ-10), metacognitive beliefs (MCQ-30), and demographic factors.

**Results:**

Maladaptive metacognitive beliefs explained additional variance in emotional distress after controlling for age, gender, time since diagnosis, physical functioning, and repetitive negative thinking. Repetitive negative thinking partially mediated the relationships between positive and negative metacognitive beliefs and emotional distress.

**Conclusions:**

These data support the utility of the metacognitive model in understanding emotional distress in people with ALS. Examination of the temporal relationship between maladaptive metacognitive beliefs and emotional distress in people living with ALS may help to guide the development of therapeutic approaches.

## Introduction

Amyotrophic lateral sclerosis (ALS) is a progressive, incurable neurodegenerative disorder affecting the motor neurones (Brooks et al., 2000). It has a reported global incidence of 1–2/100,000 person-years, with an estimated 5,000 people living with the ALS in the United Kingdom at any one time (Opie-Martin et al., 2020). The clinical presentation, rate of progression, and sequence of symptoms are variables, with gradual spreading of the disease to affect the whole body [National Institute of Neurological Disorders and Stroke (NINDS), 2013]. Key symptoms include fasciculations, muscular weakness, atrophy, dysphagia, dysarthria, and breathing difficulties (Talbot, 2009).

The average age of onset of ALS is 65 years (Talbot, 2009). ALS affects more males than females, with a lifetime risk of one in 350 for males compared with one in 472 for females in the United Kingdom (Hobson et al., 2016), but this difference diminishes with increasing age [National Institute of Neurological Disorders and Stroke (NINDS), 2013]. The life expectancy for approximately 50% of people with ALS is 3 years from symptom onset (Ilse et al., 2015), although 5–10% of people with ALS live for 10 years or more (Chiò et al., 2013).

The cause of ALS is unknown and most cases occur sporadically, although genetic risk factors account for around 5% of cases (Byrne et al., 2011). Environmental exposures, lifestyle choices, and biological ageing may contribute to causality; however, agreement about these specific factors is lacking (Al-Chalabi et al., 2010). The incidence of ALS is lower in Asia than in both Europe and North America (Aktekin and Uysal, 2020). A meta-analysis reported an incidence rate of 1.89 per 100,000 in Northern Europe, compared to 0.79 per 100,000 in South Asia (Marin et al., 2017). Demographic (e.g., density of population within at risk age), environmental, and genetic factors may all contribute to the different incidence rates (Aktekin and Uysal, 2020).

Some level of emotional distress is expected and understandable in response to a diagnosis of ALS. The unpredictable progression of ALS requires people to repeatedly adjust to new physical limitations, which understandably can impact on psychological well-being (Weeks et al., 2019). Severity of distress often oscillates, although around 50% of people with ALS experience persistent clinically significant levels of anxiety and/or depression (McDonald et al., 1994; Ganzini et al., 2002; Kübler et al., 2005). Anxiety and depression in people with ALS are associated with a poorer quality of life (Johnston et al., 1999), increased interest in hastened death (Fang et al., 2008), and elevated suicide risk (Ganzini et al., 2002). Clinical guidance recommends timely and effective psychological intervention for people with ALS experiencing depression and anxiety (National Institute for Health and Care Excellence, 2016). However, a recent systematic review concluded that “there is currently insufficient evidence to recommend the use of specific psychotherapy interventions for reducing distress or improving well-being” (pp. 293) in this patient population (Gould et al., 2015). Moreover, the psychological mechanisms that underlie anxiety and depression in ALS are poorly understood (Fisher et al., 2019a).

Arguably, efficacious interventions for people with ALS experiencing anxiety and depression must be based on a theoretical model that can accommodate the unique needs of this patient group. Traditional cognitive models suggest that the content of an individual’s thoughts, beliefs, and ideas about their health impacts upon outcomes (Becker, 1974; Rosenstock, 1974; Leventhal et al., 1984). The Common Sense Model (Leventhal et al., 1984) states that the relationship between illness perceptions and outcomes is mediated by coping strategies. Research across various health conditions has confirmed associations for aspects of this model, for example, diabetes (Lawson et al., 2010; Purewal and Fisher, 2018), stroke (Pai et al., 2019), multiple sclerosis (Bassi et al., 2020), and traumatic brain injury (Snell et al., 2013). However, a fundamental question remains – how do illness perceptions lead to the selection of coping strategies (Hagger et al., 2017)?

Cognitive behavioural therapy (CBT) aims to reduce depression and anxiety by modifying illness perceptions. Although preliminary data from a non-randomised short-term CBT trial found a reduction in depressive and anxious symptoms following a 4-session intervention (Díaz et al., 2016), CBT has not been widely or rigorously evaluated in ALS. However, wider studies indicate that CBT achieves only modest treatment effects across physical health conditions (e.g., Wolfgang, 2013; Temple et al., 2020), and likely is also of limited utility in ALS given that cognitive appraisals are often accurate; for example: “I have no control over my ALS,” “I’m not going to get better,” or “I can’t control my symptoms.” A model and associated intervention which focuses on how and why people respond to negative thoughts, such as those captured by illness perceptions, may therefore be more useful for understanding distress experienced by people with ALS.

One such model that may offer a means to understand what perpetuates distress in people with ALS is the transdiagnostic self-regulatory executive function (S-REF) model (Wells and Matthews, 1994), which underpins metacognitive therapy (MCT). The S-REF model posits that emotional distress becomes persistant when stored maladaptive metacognitive beliefs guide an individual to respond to commonly occurring thoughts and feelings in a certain way, termed the cognitive attentional syndrome (CAS). The CAS consists of: (i) perseverative thinking (e.g., worrying and overanalysing); (ii) attentional strategies (e.g., monitoring for negative thoughts and feelings); and (iii) unhelpful coping strategies (e.g., avoidance of activities). Several types of maladaptive metacognitive belief activate and sustain the CAS, resulting in emotional distress. Of particular importance are “positive” maladaptive metacognitive beliefs about the usefulness of worry (e.g., “worrying about ALS helps me to prepare for the future”) and “negative” maladaptive metacognitive beliefs about the uncontrollability and danger of worry (e.g., “I can’t control my worry about ALS”). “Positive” maladaptive metacognitive beliefs are hypothesised to indirectly cause and sustain emotional distress by increasing an individual’s likelihood of choosing to respond to thoughts with repetitive negative thinking. By contrast, “negative” maladaptive metacognitive beliefs about the uncontrollability and danger of worry are theorised to both directly and indirectly cause and sustain emotional distress. This is because these beliefs are distressing and decrease the likelihood of disengaging from repetitive negative thinking due to beliefs that thinking cannot be controlled (Wells, 2013).

The S-REF model upon which MCT is based may offer a particularly close “fit” to the needs of people with ALS for several reasons. First, the model accounts for why some people may respond to understandable negative thoughts and feelings with perseverative thinking, attentional biases and maladaptive coping strategies, whereas others may not. Second, MCT is a brief intervention which targets maladaptive metacognitive beliefs and processes. As such, it has scope to meet the specific needs and preferences of people with ALS experiencing anxiety and depression – namely, to help reduce distressing levels of worry and rumination and reduce coping strategies which can exacerbate distress (Weeks et al., 2019).

There is growing support for the clinical applicability of the S-REF model to emotional distress in physical health conditions (Capobianco et al., 2020)–specifically, for people with cancer (Butow et al., 2015; Cook et al., 2015a,b), diabetes (Purewal and Fisher, 2018), epilepsy (Fisher and Noble, 2017; Fisher et al., 2018), multiple sclerosis (Heffer-Rahn and Fisher, 2018), chronic fatigue syndrome (Maher-Edwards et al., 2011), fibromyalgia (Kollmann et al., 2016), cardiovascular disease (Anderson et al., 2019), and Parkinson’s disease (Brown and Fernie, 2015). Preliminary evidence indicates that MCT may be an effective and tolerable intervention for adults with physical health difficulties (Cherry et al., 2019; Fisher et al., 2019b; McPhillips et al., 2019). However, the utility of the S-REF model in understanding emotional distress in people with ALS has not been studied. As a first step toward developing more efficacious interventions for people with ALS, this study investigates the relationships between metacognitive beliefs, repetitive negative thinking and anxiety and depression in people with ALS. After controlling for clinical and demographic factors, it is hypothesised that:

1. Maladaptive metacognitive beliefs will explain additional variance in emotional distress (anxiety and depression) over and above that explained by repetitive negative thinking.
2. The relationship between “positive” maladaptive metacognitive beliefs and emotional distress will be fully mediated by repetitive negative thinking.
3. The relationship between “negative” maladaptive metacognitive beliefs and emotional distress will be partially mediated by repetitive negative thinking.

## Materials and Methods

### Design

The study adopted a cross-sectional design with multiple self-report measures.

### Participants

**FIGURE 1:**
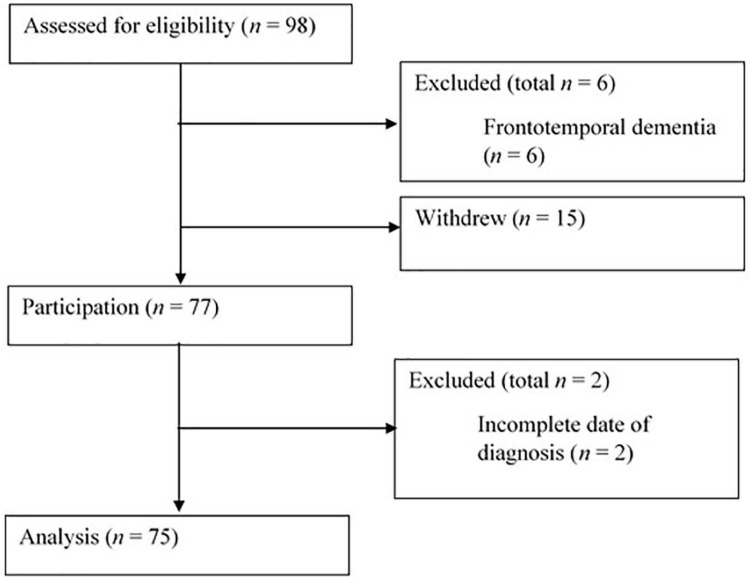
Participant flow diagram.

Convenience sampling methods were utilised, with participants recruited from clinic appointments, charity branch meetings, social media, and poster advertisements. Participants were 75 adults who met the following inclusion criteria: (i) provided informed written consent; (ii) a self-reported diagnosis of ALS; (iii) a self-reported absence of frontotemporal dementia; (iv) ability to understand written English; (v) aged 18 years or over; and (vi) completion of all study measures. Figure 1 displays the flow of participants through the study.

### Measures

#### Demographic and Clinical Information

A self-report questionnaire assessed age, gender, date of diagnosis, date of symptom onset, current medications, and co-morbid health conditions.

#### Emotional Distress

Emotional distress was assessed using a version of the Hospital Anxiety and Depression Scale (HADS) (Zigmond and Snaith, 1983) adapted for people with ALS (Gibbons et al., 2011). The adapted HADS is a 11-item self-report questionnaire in which participants rate the presence of symptoms of anxiety (six items) and depression (five items) over the preceding week using a 4-point Likert scale, with options from 0 (absence) to 3 (extreme presence). Responses can be summed to produce a total emotional distress score (HADS-T), ranging from 0 to 33, with higher scores indicative of higher emotional distress levels. The adapted HADS demonstrates acceptable internal consistency in an ALS population (PSI = 0.86; Gibbons et al., 2011). Cronbach’s alpha for the adapted HADS in the current study was 0.91.

#### Metacognitive Beliefs

Maladaptive metacognitive beliefs were assessed using the Metacognitions Questionnaire-30 (MCQ-30; Wells and Cartwright-Hatton, 2004). The MCQ-30 is a 30-item self-report measure which assesses five maladaptive metacognitive belief domains: (i) “positive” metacognitive beliefs; (ii) “negative” metacognitive beliefs; (iii) cognitive confidence; (iv) need to control thoughts; and (v) cognitive self-consciousness. Respondents rate whether they “generally agree” with each statement using a 4-point Likert scale, with options extending from 1 (“do not agree”) to 4 (“agree very much”). Responses can be summed to produce five subscale scores (ranging from six to 24) and a total score (ranging from 30 to 120), with higher scores indicating higher levels of unhelpful metacognitions. The MCQ-30 subscales demonstrate adequate internal consistency (α = 0.72–0.89; Wells and Cartwright-Hatton, 2004) and have been used in a range of physical health populations (Cook et al., 2014; Fisher and Noble, 2017; Fisher et al., 2018; Purewal and Fisher, 2018). Cronbach’s alphas for the subscales in the current study ranged from 0.66 to 0.91.

#### Repetitive Negative Thinking

Repetitive negative thinking was assessed using the trait version of the Repetitive Thinking Questionnaire (RTQ-10; McEvoy et al., 2014). The RTQ-10 is a 10-item self-report measure in which participants rate their likelihood of responding in a certain way to distressing situations using a 5-point Likert scale, with options that extend from 1 (“not true at all”) to 5 (“very true”). Responses are summed to produce a total score, ranging from 10 to 50, with higher scores indicating higher levels of repetitive negative thinking. The RTQ-10 demonstrates acceptable internal consistency (α ≥ 0.89; McEvoy et al., 2010). Cronbach’s alpha in the current study was 0.92.

#### Physical Functioning

Physical functioning was assessed using the Self-Administered ALS Functional Rating Scale – Revised (ALSFRS-R; Montes et al., 2006). The ALSFRS-R is a 12-item self-report measure designed to assess physical functioning, including bulbar, limb, and respiratory function related to activities of daily living, in people with ALS. Participants rate current function compared to pre-diagnostic function on a 4-point Likert scale, with options extending from 0 (“unable to do”) to 4 (“no change”). Responses are summed to produce a total score, ranging from 0 to 48, with higher scores indicating better physical functioning. The scale has satisfactory internal consistency (α = 0.73; Cedarbaum et al., 1999); Cronbach’s alpha for the current study was 0.87.

### Procedure

Ethical approval was obtained from the Liverpool Central Health Research Authority research and ethics committee (Reference:16/NW/0073). Participants were recruited: (i) from outpatient clinics at two National Health Service (NHS) hospitals in the North of England; (ii) from branch meetings of the Motor Neurone Disease Association (MND Association), a United Kingdom charity for people with ALS; and (iii) online *via* social media platforms of third sector organisations providing support to people with ALS. For the NHS sites, eligible patients attending outpatient clinic appointments were approached by a member of their clinical team and asked to participate. Additionally, posters were placed in clinics inviting interested patients to speak to the researcher or their clinical team about the study. Eligible and interested individuals could either complete and return a study pack directly or participate online. Participants could choose whether to complete the measures alone, with researcher support, or with support from another trusted person. Regardless of recruitment method, all participants were asked to read the participant information sheet, provide informed consent and complete the study measures, which were presented in a random order to participants to prevent order effects.

### Statistical Analysis

Raw data were first screened for inputting errors and summed scale scores were calculated where appropriate. Missing data were deemed to be missing at random, and therefore the one missing value from the MCQ-30 was managed using mean imputation (Raymond, 1986) which has demonstrated superiority in estimating parameters (Dodeen, 2003). Data were checked for normality of distribution; several variables showed evidence of skewness, and therefore, correlational analyses and Mann–Whitney *U* tests were used as appropriate for initial data exploration. Hierarchical linear regression was then used to test the hypothesis that maladaptive metacognitive beliefs would explain additional variance in emotional distress after controlling for basic demographics, physical functioning, and repetitive negative thinking. Predictor variables were entered in the following order: Step 1–age, gender; Step 2–time since diagnosis, physical functioning; Step 3–repetitive negative thinking; Step 4–maladaptive metacognitive beliefs (all subscales). The fit of data within the assumptions of multiple linear regression was assessed by examining the distribution and heteroscedasticity of regression residuals; no violations were identified. Finally, to test the hypothesised relationships between maladaptive metacognitive beliefs, repetitive negative thinking and emotional distress, two mediational analyses were conducted (Model 1: *x* = “positive” maladaptive metacognitive beliefs, *m* = repetitive negative thinking, *y* = emotional distress; Model 2: *x* = “negative” maladaptive metacognitive beliefs, *m* = repetitive negative thinking, *y* = emotional distress). In both models, age, gender, physical functioning, and time since diagnosis were controlled for. Bootstrapped (5,000 samples) bias-corrected and accelerated (BCa) estimates, and 95% confidence intervals are reported for the indirect effect (Preacher and Hayes, 2008). All analyses were conducted in Statistical Package for Social Sciences (SPSS version 22), with the PROCESS macro (Hayes, 2013) used for mediational analyses; *p* values of < 0.05 were considered statistically significant throughout.

### Sample Size, Power, and Precision

An *a priori* power calculation indicated that, to adequately detect a medium to large effect size (*f*^2^ = 0.25) with a 0.80 power level and a standard α level of 0.05, a minimum of 75 participants were required for the most complex planned analysis: a multiple linear regression containing 10 predictor variables (Faul et al., 2009).

## Results

### Participant Demographics

Table 1 displays participants’ demographic characteristics. Most participants were male, with a mean age of 60.40 years. Two participants were receiving nutrition *via* percutaneous endoscopic gastrostomy. Two required intubation or tracheotomy, sixteen required non-invasive ventilation: eight during the day and night, six during the night only, and two intermittently. Of the sample, 22.10% met the criteria for severe depression and 19.48% for severe anxiety (scores of eight and above on adapted HADS-D and nine and above on adapted HADS-A respectively; Gibbons et al., 2011). Thirty six participants were treated with Riluzole and 25 were prescribed an antidepressant, however, it was unclear whether this was to treat depression or to manage symptoms of MND such as hypersalivation through its side effects.

**TABLE 1:** Demographic data (*n* = 75).

**Demographic variable**	***n* (%), Unless otherwise stated**
Age (years)	60.40 (10.72), 29–86^*a*^
**Gender**	
Male	42 (56.00)
Female	33 (44.00)
Time since onset of symptoms (months)	63.92 (64.78), 5–351^*a*^
Time since diagnosis (months)	43.61 (52.19), 1–258^*a*^
Additional health difficulties	47 (62.70)
**Medication**	
No medication	13 (17.30)
Riluzole	23 (30.70)
Antidepressant	12 (16.00)
Riluzole and antidepressant	13 (17.30)
Other	14 (18.70)

*^*a*^Mean (standard deviation) and range.*

### Preliminary Data Analysis and Hypothesis Testing

As expected, time since diagnosis significantly negatively correlated with ALSFRS-R score (*r* = −0.36). Age was significantly correlated with cognitive confidence (*r* = 0.28). There was also a significant effect of gender on cognitive confidence, with males (*Mdn* = 9.50) scoring higher than females (*Mdn* = 7.00), *U* = 524.50, *z* = −2.20, *p* = 0.03, *r* = −0.25. Table 2 displays inter-correlations and descriptive statistics for the main independent (MCQ-30 subscales) and dependent (HADS-T) variables. As expected, emotional distress was significantly positively correlated with three maladaptive metacognitive belief domains: “negative” maladaptive metacognitive beliefs about the uncontrollability and danger of worry (*r* = 0.68), cognitive confidence (*r* = 0.26), and cognitive self-consciousness (*r* = 0.36). Furthermore, emotional distress was also positively correlated with repetitive negative thinking (*r* = 0.69). Repetitive negative thinking was significantly positively correlated with the majority of the MCQ subscales, apart from cognitive confidence (*r* values range from 0.24 to 0.75).

**TABLE 2:** Descriptive statistics and Spearman’s rho correlations between study variables.

	**2**	**3**	**4**	**5**	**6**	**7**	***Mdn***	***IQR***
1) HADS-T	0.69***	0.14	0.68***	0.26*	0.06	0.36**	9.00	5.00–14.00
2) RTQ-10	–	0.37**	0.75***	0.19	0.24*	0.49***	24.00	16.00–33.00
3) MCQ-PB		–	0.31**	0.14	0.29*	0.28*	7.00	6.00–11.00
4) MCQ-NB			–	0.12	0.21	0.57***	10.00	6.50–14.00
5) MCQ-CC				–	0.26*	0.01	8.00	6.00–11.00
6) MCQ-NC					–	0.36**	9.00	8.00–12.00
7) MCQ-CSC						–	14.00	11.00–16.50

HADS-T, adapted Hospital Anxiety and Depression Scale total score; IQR, interquartile range; MCQ-CC, Metacognitions Questionnaire-30 cognitive confidence subscale; MCQ-CSC, Metacognitions Questionnaire-30 cognitive self-consciousness subscale; MCQ-NB, Metacognitions Questionnaire-30 negative beliefs about the uncontrollability and danger of worry subscale; MCQ-NC, Metacognitions Questionnaire-30 need for control subscale; MCQ-PB, Metacognitions Questionnaire-30 positive beliefs about worry subscale; Mdn, median; RTQ-10, Repetitive Thinking Questionnaire. *p < 0.05, **p < 0.01, and ***p < 0.001.

### Main Hypothesis Testing

#### Regression Analysis

The results of the regression analysis are shown in Table 3.

**TABLE 3:** Hierarchical multiple linear regression model predicting emotional distress.

**Variable**		**Δ*R*^2^**	**Δ*F***	***b***	**β**	**95% BCa CIs**
Step 1	Age Gender	0.01	0.30	−0.04 0.59	−0.07 0.05	−0.16 to 0.08 −2.43 to 3.86
Step 2	Age Gender ALSFRS-R Time since dx	0.03	1.01	−0.05 0.60 −0.11 −0.01	−0.08 0.05 −0.17 −0.01	−0.17 to −0.75 −2.46 to 3.81 −0.28 to 0.05 −0.03 to 0.03
Step 3	Age Gender ALSFRS-R Time since dx RTQ-10	0.43	56.24***	0.04 2.12 −0.06 <−0.01 0.43	0.06 0.16 −0.09 −0.01 0.69***	−0.06 to 0.14 −0.22 to 4.33 −0.17 to 0.04 −0.02 to 0.02 0.29 to 0.57
Step 4	Age Gender ALSFRS-R Time since dx RTQ-10 MCQ-PB MCQ-NB MCQ-CC MCQ-NC MCQ-CSC	0.12	3.72***	0.02 1.27 −0.04 <0.01 0.30 −0.33 0.55 0.11 −0.35 0.06	0.03 0.10 −0.07 0.02 0.48*** −0.20* 0.38** 0.06 −0.17 0.04	−0.08 to 0.13 −0.93 to 3.28 −0.16 to 0.07 −0.02 to 0.03 0.12 to 0.50 −0.64 to 0.03 0.14 to 0.92 −0.26 to 0.61 −0.69 to 0.14 −0.29 to 0.32

ALSFRS, Amyotrophic Lateral Sclerosis Functional Rating Scale – Revised; dx, diagnosis; MCQ-CC, Metacognitions Questionnaire-30 cognitive confidence subscale; MCQ-CSC, Metacognitions Questionnaire-30 cognitive self-consciousness subscale; MCQ-NB, Metacognitions Questionnaire-30 negative beliefs about the uncontrollability and danger of worry subscale; MCQ-NC, Metacognitions Questionnaire-30 need for control subscale; MCQ-PB, Metacognitions Questionnaire-30 positive beliefs about worry subscale; RTQ-10, Repetitive Thinking Questionnaire. *p < 0.05, **p < 0.01, and ***p < 0.001. There was no evidence of multicollinearity or autocorrelation (variance inflation factors < 10, tolerance statistics > 0.2, correlations between study variables < 0.8, Durbin–Watson test statistic of 2.18).

Hypothesis 1: Maladaptive metacognitive beliefs will explain additional variance in emotional distress (anxiety and depression) over and above that explained by repetitive negative thinking.

After controlling for age, gender, and ALS characteristics, repetitive negative thinking accounted for an additional 43% of the variance in emotional distress. Addition of maladaptive metacognitive belief domains explained an additional 12% of the variance in emotional distress. The final model for emotional distress accounted for 59% of the variance [adjusted *R*^2^ = 0.59, *F* (10, 74) = 9.15, *p* < 0.01]. Repetitive negative thinking and two specific maladaptive metacognitive belief domains (positive beliefs about worry and negative beliefs about the uncontrollability and danger of worry) made independent contributions to the model.

#### Mediation of the Association Between Metacognitive Beliefs and Emotional Distress by Repetitive Negative Thinking

**FIGURE 2:**
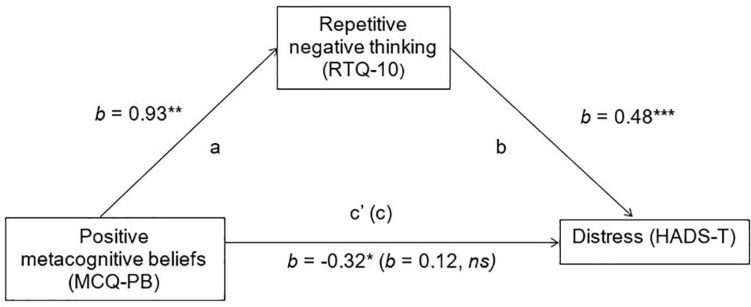
Mediation of positive met a cognitive beliefs on emotional distress, *via* Repetitive Negative Thinking. HADS-T, total adapted Hospital Anxiety and Depression Scale score; MCQ-PB, Metacognitions Questionnaire-30 positive beliefs about worry subscale; RTQ-10, Repetitive Thinking Questionnaire; ns, non-significant. *p < 0.05_:_ **p < 0.01, and ***p < 0.001.

**FIGURE 3:**
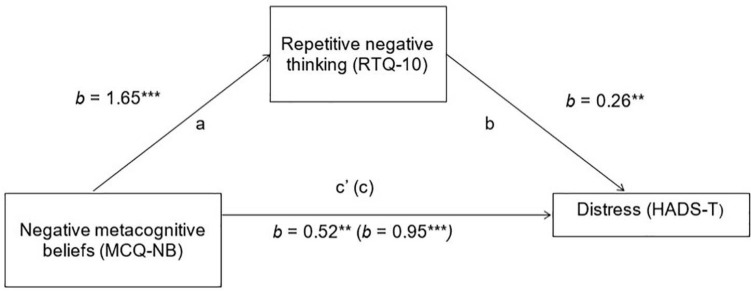
Mediation of negative metacognitive beliefs on emotional distress, *via* Repetitive Negative Thinking. HADS-T, total adapted Hospital Anxiety and Depression Scale score; MCQ-NB, Metacognitions Questionnaire-30 negative beliefs about worry subscale; RTQ-10, Repetitive Thinking Questionnaire. *p < 0.05, **p < 0.01, and ***p < 0.001.

Results of the mediation analyses are shown in Figures 2, 3. Age, gender, time since diagnosis, and physical functioning (ALSFRS-R scores) were controlled for in both mediation models.

Hypothesis 2: The relationship between “positive” maladaptive metacognitive beliefs and emotional distress will be fully mediated by repetitive negative thinking.

In the mediation model assessing the role of repetitive negative thinking in mediating the relationship between positive’ maladaptive metacognitive beliefs and emotional distress, there was a significant indirect effect of repetitive negative thinking (ab = 0.44, BCa 95% CIs = 0.19–0.81). There was no direct effect, indicating full mediation. However, it should be noted that there was a negative relationship between positive’ maladaptive metacognitive beliefs and emotional distress.

Hypothesis 3: The relationship between “negative” maladaptive metacognitive beliefs and emotional distress will be partially mediated by repetitive negative thinking.

There was also a significant indirect effect in the mediation model assessing repetitive negative thinking as a mediator of the relationship between “negative” maladaptive metacognitive beliefs about the uncontrollability and danger of worry and emotional distress (ab = 0.42, BCa 95% CIs = 0.10–0.82). As with the first model, the direct effect remained significant, indicating partial mediation.

## Discussion

This was the first study to examine the association between maladaptive metacognitive beliefs, repetitive negative thinking, and emotional distress in an ALS population, with the aim of testing predictions derived from the S-REF model. As hypothesised, maladaptive metacognitive beliefs accounted for an additional 12% of the variance in emotional distress after controlling for age, gender, time since diagnosis, physical functioning, and repetitive negative thinking. Moreover, only repetitive negative thinking, “negative” maladaptive metacognitive beliefs about the danger, and uncontrollability of worry and “positive” maladaptive metacognitive beliefs about worry made significant contributions to the final model. This supports previous research indicating that maladaptive metacognitive beliefs are associated with emotional distress in individuals with physical health difficulties (Brown and Fernie, 2015; Cook et al., 2015a,b; Fisher and Noble, 2017; Heffer-Rahn and Fisher, 2018; Purewal and Fisher, 2018) and reflects previous findings that physical functioning, time since diagnosis, and demographic factors are inconsistent predictors of emotional distress in people with ALS (De groot et al., 2007; Gauthier et al., 2007; Cupp et al., 2011; Matuz et al., 2015; Fisher et al., 2019b).

Mediational analyses largely supported the hypothesised relationships between maladaptive metacognitive beliefs, repetitive negative thinking and emotional distress. As expected, the relationship between “negative” maladaptive metacognitive beliefs and emotional distress was partially mediated by repetitive negative thinking, which is consistent with both the main thesis of the S-REF model and the findings of previous research (Cook et al., 2015a,b; Heffer-Rahn and Fisher, 2018). However, the relationship between “positive” maladaptive metacognitive beliefs and emotional distress was only partially mediated by repetitive negative thinking. Furthermore, “positive” maladaptive metacognitive beliefs were negatively associated with emotional distress. Although this supports the thesis that “positive” maladaptive metacognitive beliefs act indirectly to cause and sustain emotional distress by promoting the use of worry and rumination as maladaptive coping strategies, the negative relationship between “positive” maladaptive metacognitive beliefs is not clearly understood. It may be that the perceived helpfulness of thinking about difficulties provides a sense of control and ability to cope in the short-term, therefore reducing emotional distress (Hayes et al., 2006). However, when “positive” maladaptive metacognitive beliefs lead to the selection of persistent repetitive negative thinking as a coping strategy, this results in emotional distress. This is clearly an area that would, benefit from further research.

## Limitations and Future Research Directions

There are several limitations to this study which may influence the generalisability of findings. First, the study was cross-sectional and therefore causality cannot be assumed. Second, consideration of unmeasured variables, such as social support or psychological support (Goldstein et al., 2006; Matuz et al., 2015), may have resulted in a more robust model that was able to account for a larger proportion of the variance.

Third, various adjustments were made to improve accessibility for patients in a hard to reach group, which limited the reliability and detail of data collected. Given these encouraging initial findings, tests of the S-REF model could be applied to other forms of emotional distress e.g., traumatic stress symptoms. The S-REF model could be tested in its entirety e.g., through the including assessment of threat monitoring e.g., feelings of distress and presence of negative thoughts (illness perceptions). People with comorbid physical health conditions and clinical levels of emotional distress often using counterproductive coping strategies (e.g., trying not to have negative thoughts, thinking positively, or trying to avoid situations or events which may trigger worry and rumination). All these domains should be assessed in future tests of the S-REF model, as well as objectively verifying key clinical variables such as frontotemporal dementia and diagnosis of ALS. Objective verification is particularly important for frontotemporal dementia diagnosis, given altered insight into deficits is a key clinical feature (Muñoz-Neira et al., 2019).

Finally, although all analyses were adequately powered (*post hoc* power for both final regression models = 0.80; *p* = 0.05), the sample size was modest and may not generalise to the ALS population. There were few individuals receiving nutrition *via* percutaneous endoscopic gastrostomy or required assisted ventilation and there was a greater duration of disease, compared to the general ALS population (Ilse et al., 2015). Future testing of the model in a larger, more representative sample of people with ALS would allow for more detailed testing of the S-REF model through subgroup analyses (e.g., grouping individuals based on time since diagnosis or site of onset). However, it should be recognised that recruiting and retaining people with ALS with severe symptoms in research studies can be challenging given poor health and prioritisation of end of life needs (Albert et al., 2005).

To translate interventions, longitudinal multi-site designs are required to test the causal role of metacognitive beliefs in emotional distress experienced by people with ALS. Despite the difficulties associated with conducting longitudinal research in this population, consideration of ALS-specific factors could further our understanding of the S-REF model in this population. These may include the relationship of perceived ability to communicate effectively with the level of repetitive negative thinking or the influence of social cognition change on theory of mind and emotional distress (van der Hulst et al., 2015). Such research could potentially support identification and implementation of support for those at risk of developing emotional distress. Future research should also take a between-group approach and consider how metacognitive beliefs present in those who adapt well to ALS, how they respond to thoughts, the coping strategies this leads them to adopt, and how this impacts on their psychological wellbeing. Examples of adaptive metacognitive beliefs in this population might include “I can choose whether to engage with my thoughts about ALS,” and “I can manage my worries, they will pass.” The S-REF model posits that these beliefs would result in an ability to disengage from thoughts and subsequently lowered distress levels; this would be a valuable hypothesis to explore further.

## Conclusion

In summary, our findings indicate that maladaptive metacognitive beliefs and repetitive negative thinking are strongly associated with emotional distress in people with ALS and account for substantial variance in emotional distress and after controlling for demographic and clinical factors. This is in keeping with findings of other studies examining the fit of the S-REF model in physical health populations (Capobianco et al., 2020). If tailored to the needs of people with ALS, MCT could be an acceptable psychological intervention. Better understanding of metacognitive beliefs and processes in this population may provide a basis for investigating the efficacy of MCT for reducing depression and anxiety in people with ALS.

## Data Availability Statement

The raw data supporting the conclusions of this article will be made available by the authors, without undue reservation.

## Ethics Statement

The studies involving human participants were reviewed and approved by the Liverpool Central Health Research Authority Research and Ethics Committee (reference: 16/NW/0073). The patients/participants provided their written informed consent to participate in this study.

## Author Contributions

PF conceptualised the study and supervised its conduct. RD collected the data and drafted the initial manuscript. PM and SM co-supervised the conduct of the study. MC assisted with data analysis and write-up. All authors read and commented on a draft of the manuscript.

## Conflict of Interest

The authors declare that the research was conducted in the absence of any commercial or financial relationships that could be construed as a potential conflict of interest.
